# Coastal bathymetry in central Dronning Maud Land controls ice shelf stability

**DOI:** 10.1038/s41598-024-51882-2

**Published:** 2024-01-16

**Authors:** H. Eisermann, G. Eagles, W. Jokat

**Affiliations:** Alfred Wegener Institute for Polar and Marine Research, Am Alten Hafen 26, Bremerhaven, Germany

**Keywords:** Geophysics, Cryospheric science

## Abstract

Knowledge of the bathymetry of Antarctica’s margins is crucial for models and interpretations of ice-ocean interactions and their influence on ongoing and future sea level change, but remains patchy where ice shelves and multi-year sea ice block measurements. Here, we present a bathymetric model for the central Dronning Maud Land margin, based on a constrained inversion of airborne gravity data. It shows the cavities beneath the region’s two ice shelves to be much deeper than in existing bathymetric compilations, but to be shielded from Warm Deep Water ingress and basal melting by the presence of shallow bathymetric sills along the continental shelf. Over areas of multi-year sea ice, the model returns bathymetric estimates of similar accuracy to gravity interpolation-based methods over open water. Airborne gravity thus presents an opportunity to bathymetrically map hundreds of thousands of square kilometres of the most inaccessible margins of Antarctica at resolutions adequate for regional and global oceanographic and glaciological modelling and interpretation.

## Introduction

Floating ice shelves buttress the majority of Antarctica’s grounded ice, making them a key factor for its stability in a warming climate. The systems of ice shelves and ice sheets are connected in such a way that net ice mass losses from ice shelves, due to iceberg calving or surface and basal melting processes, lead to increased drainage of their corresponding ice sheets^1,2^. If this is not counterbalanced by mass gain processes, such as basal refreezing or snowfall, the net mass loss will subsequently contribute to global sea level rise. The processes leading to ice mass gains and losses must therefore be evaluated with the best precision to assess the stability of an ice shelf-ice sheet system.

The mass loss process of basal melting dominates Antarctica’s ice shelves^3^ and has proven to be highly dependent on water temperatures in the cavities beneath them^4^ and their underlying bathymetry^5,6^. Bedrock topography not only controls the circulation of water within a cavity between seabed and ice base, but is also a deciding factor on whether and where warm oceanic water residing at intermediate depths along the coast breaches into that cavity^7–9^. Offshore, bathymetry influences current steering and upwelling patterns and, consequently, the overall oceanographical setting at the coast. Consistent bathymetric models of the Antarctic margins are thus vital to improve our understanding of polar aspects of the Earth system by numerical modelling.

Sub-metre accuracy and metre-scale resolution in such data can be achieved by direct soundings. Unfortunately, the majority of the ice shelf cavities and much of the open ocean directly encircling the Antarctic margin remain unexplored in this way, as outlined by the RINGS Action Group^10^. Logistical constraints and costly survey platforms limit the availability of topographic and bathymetric measurements underneath and in front of ice shelves. Over the open oceans, bathymetry can additionally be estimated by methods that take advantage of its correlation to sea-surface heights, which can be measured by satellite radar altimetry, achieving accuracies in the level of 150–180 m for grid cells of a few kilometres in size^11^. Close to the Antarctic coasts, however, multi-year sea ice strongly degrades the quality of such estimates, preventing them from being included in current compilations of Antarctic topography and bathymetry^12^. Topographic compilations of Antarctica and the Southern Ocean, such as Bedmap^13,14^, BedMachine Antarctica^15^, or IBCSO^12^ continue thus to present huge areas of the Antarctic margins with bathymetric information derived by interpolation methods alone.

**Figure 1:**
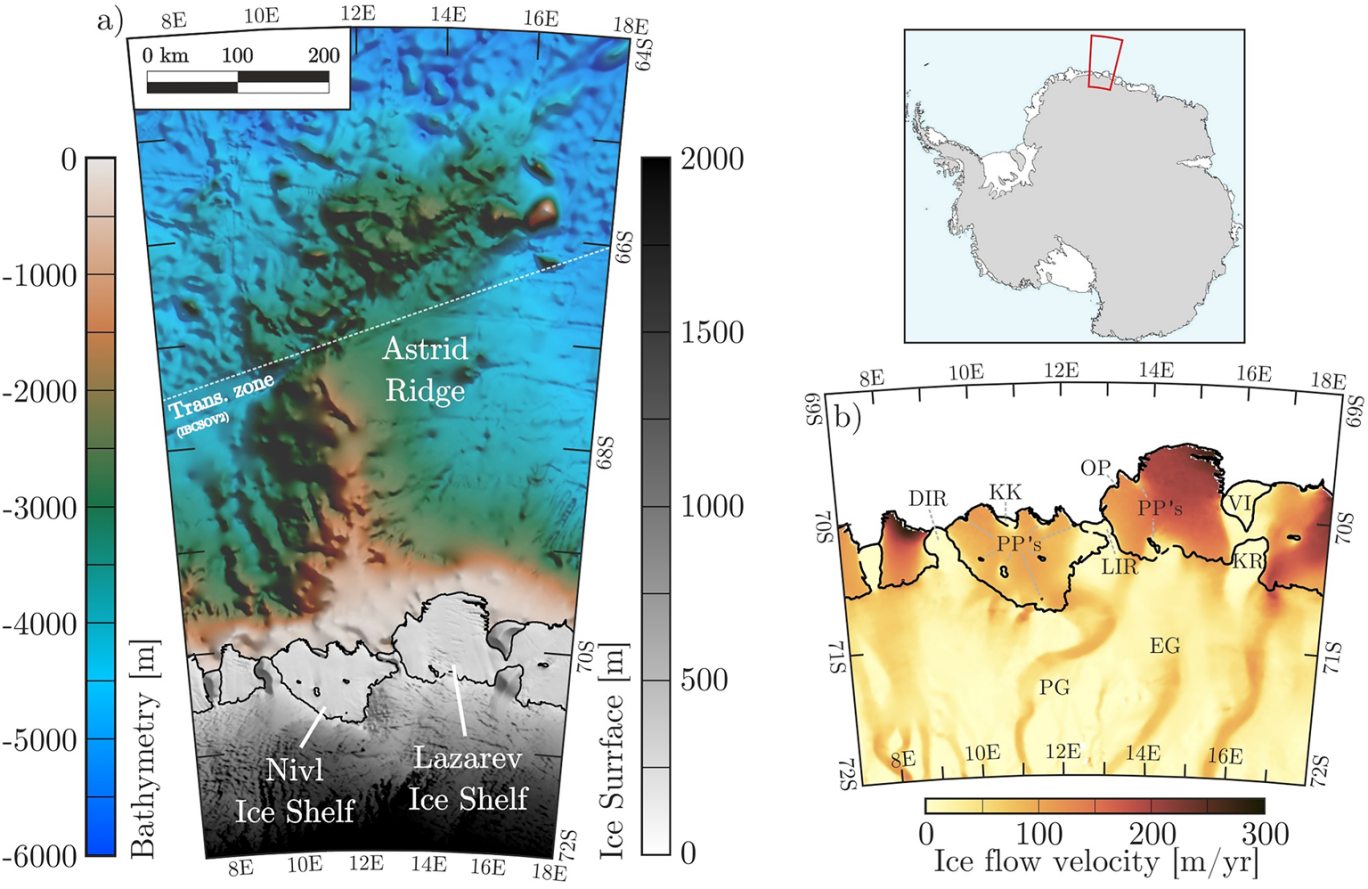
The central Dronning Maud Land sector of the East Antarctic margin, with the Nivl and Lazarev ice shelves and offshore Astrid Ridge. (**a**) Seabed depths from IBCSO V2^12^ overlain by the digital elevation model REMA^16^ in ice-covered regions. Northwards of the white dashed line, IBCSO V2 incorporates bathymetry derived by correlation to satellite altimetry^11^; southwards of the line, the IBCSO V2 compilation does not include it and marks it as a ‘transition zone’ (Trans. zone). (**b**) Ice flow velocities^17^ in the survey area. Here, names of glaciers (EG: Entuziasty Glacier; PG: Potsdam Glacier), ridges (KR: Kamelryggen), ice rises (DIR: Djupranen Ice Rise; LIR: Leningradkollen Ice Rise), islands (VI: Verblyud Island), and pinning points (KK: Kuvklaken; OP: Opornyy Point) are given. Additional unnamed pinning points are marked with PP’s for both ice shelves. Calving fronts and grounded areas including pinning points are extracted from MEaSUREs data collection^18^. Figure is generated with *Seequent’s Geosoft Oasis montaj* and *Corel Draw*.

To fill part of this vast bathymetric knowledge gap, we complement existing bathymetric information in central Dronning Maud Land using airborne gravity data measurements. Unlike depths estimated from sea-surface height, the accuracy of these data is not affected by the presence of floating ice (i.e., sea ice and ice shelves in hydrostatic equilibrium). To minimize the effects of the inherent non-uniqueness of gravity models, we use the existing topographic information from the Nivl Ice Shelf, the Lazarev Ice Shelf, and the Astrid Ridge offshore (Fig. 1) to constrain an inversion of airborne gravity data across the survey area. The newly generated model extends current topographic knowledge in the region, allowing us to interpret it in terms of glacial history and potential ongoing ice-ocean interactions.

### Setting at Nivl and Lazarev ice shelves, Astrid Ridge

The Nivl and Lazarev ice shelves extend from 69° to 71° South and from 9° to 16.5° East along the coast of Dronning Maud Land (Fig. 1) and cover areas of 7285 km^2^ and 8519 km^2^ respectively^3^. While the Leningradkollen Ice Rise separates the Nivl and Lazarev ice shelves, the Nivl is delimited by the Djupranen Ice Rise in the west and the Lazarev is confined by the Verblyud Island and the Kamelryggen in its east (Fig. 1b). The Nivl Ice Shelf is grounded at pinning points distributed latitudinally along lines passing through its midpoint and along the calving front, with only one named as Kuvklaken (Fig. 1b). The Lazarev Ice Shelf is grounded by pinning points close to its grounding line and two pinning points at its calving front, one named Opornyy Point (Fig. 1b). The Potsdam Glacier feeds the Nivl Ice Shelf via four branches and the Lazarev Ice Shelf by one^19^. The Lazarev Ice Shelf is additionally linked to the Entuziasty Glacier. Across the ice shelves, the ice flow is channelled between the pinning points (Fig. 1b).

Offshore, Dronning Maud Land’s otherwise-narrow continental shelf^12,20^ widens because of the presence of the Astrid Ridge (Fig. 1a), a broad spur of thickened oceanic crust that separates the western Riiser-Larsen Sea from the eastern Lazarev Sea. It formed during Gondwana breakup in Jurassic times^21,22^. The ridge extends northwards, narrowing and bending slightly towards the east until it terminates at ~ 65° S (Fig. 1a).

Water masses off coastal Dronning Maud Land are shaped by the Antarctic Circumpolar Current and the local Weddell Gyre. Warm and salty Circumpolar Deep Water, transported by the Antarctic Circumpolar Current, intrudes the Weddell Gyre at its eastern fringe and flows towards the west as Warm Deep Water along the continental rise and slope of Dronning Maud Land^23^. At present, the cavities on the shelf mainly host cold Eastern Shelf Water with regional and sporadic intrusion of Warm Deep Water, largely controlled by topographic features^7,8,24^. Seasonal intrusions of solar-heated Antarctic Surface Water with subsequent increases in basal melt rates have been observed recently for the Nivl Ice Shelf^25^ and its western neighbour, the Fimbul Ice Shelf^7,26^. Basal melting processes account for about three quarters of the mass loss from both the Nivl and Lazarev ice shelves^3^, marking them as highly unconventional amongst the cold-cavity ice shelves of Dronning Maud Land. Despite the predominance of mass loss by basal melting in this area, Warm Deep Water is assumed not to play a large role, at least for the Nivl Ice Shelf^25^, owing to the shallow bathymetry observed, albeit patchily, along the calving front.

### Available data and gravity inversion

Our bathymetry model is based on available topographic data sets and the inversion of airborne gravity data, similar to studies^6,24^. The reliability of these models is highly dependent on the abundance and quality of independent bathymetric and topographic data that constrain the inversion procedure.

**Figure 2:**
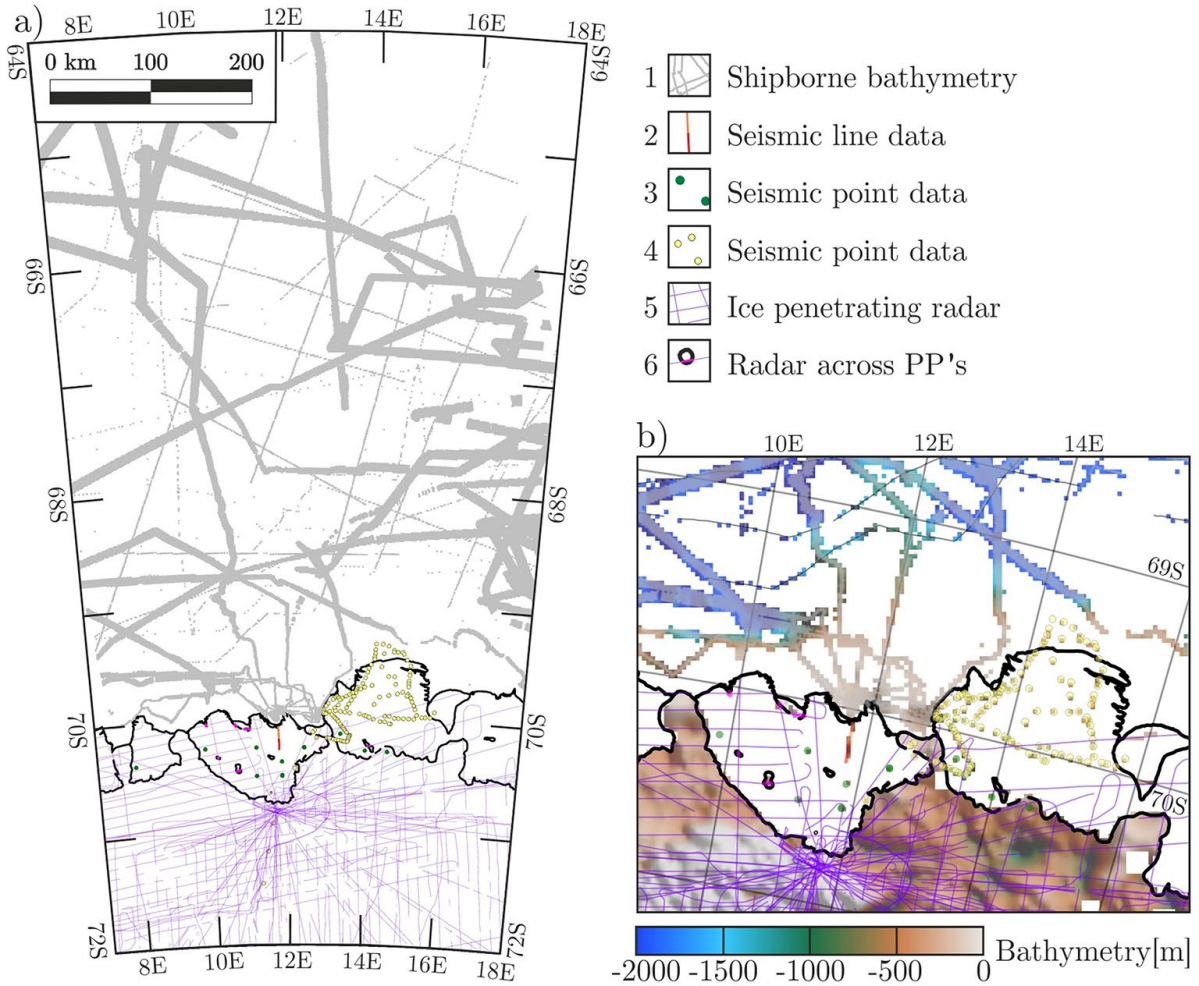
Bathymetric and topographic constraints for Astrid Ridge and the Nivl and Lazarev ice shelves. (**a**) Topographic data sets consisting of shipborne hydroacoustic data^12^ (1), seismic reflection line data (2, Fig. 3, M. Degutsch, pers. comm.), seismic point data^14,33,34^ (3, 4), and ice penetrating radar data across the survey area^32^ (5) and above pinning points^25,32^ (PP’s; 6). (**b**) shows known bedrock topography derived from available data and its sources^12,14,25,32–34^ in (**a**). Calving fronts and grounded areas are extracted from MEaSUREs data collection^18^. Figure is generated with *Seequent’s Geosoft Oasis montaj* and *Corel Draw*.

**Figure 3:**
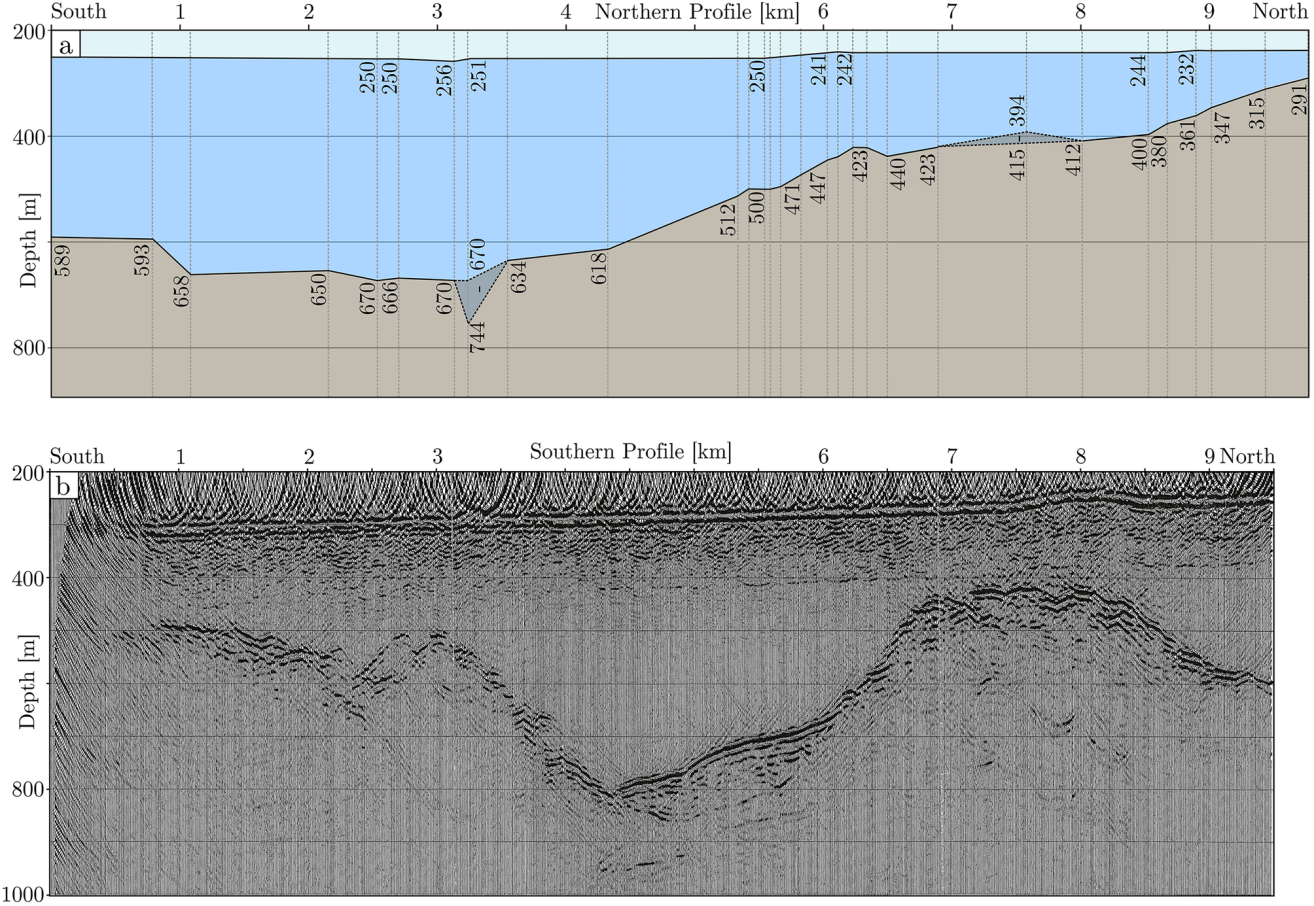
Seismic profile along the Nivl Ice Shelf. Profile is segmented in (**a**) a single-fold northern line (modified from ref.^35^) with marked depths and (**b**) a ten-fold southern profile, which has undergone deconvolution and migration (modified from ref.^36^). Vertical exaggeration for both panels is 4.2. Figure is generated with *Corel Draw*.

Available topographic data stem from a variety of sources across the survey area (Fig. 2). Shipborne hydroacoustic measurements in areas of open ocean^12,27–31^ are combined with ice penetrating radar data^32^, and seismic reflection data^33^, including a previously unpublished seismic reflection line across the north-eastern Nivl Ice Shelf (Fig. 3; Table S1 in Supplementary Information; M. Degutsch, pers. comm.). After compiling the available topographic data, however, large gaps of up to 100 × 100 km remain across the Astrid Ridge and 50 × 50 km for the Nivl and Lazarev ice shelves.

**Figure 4:**
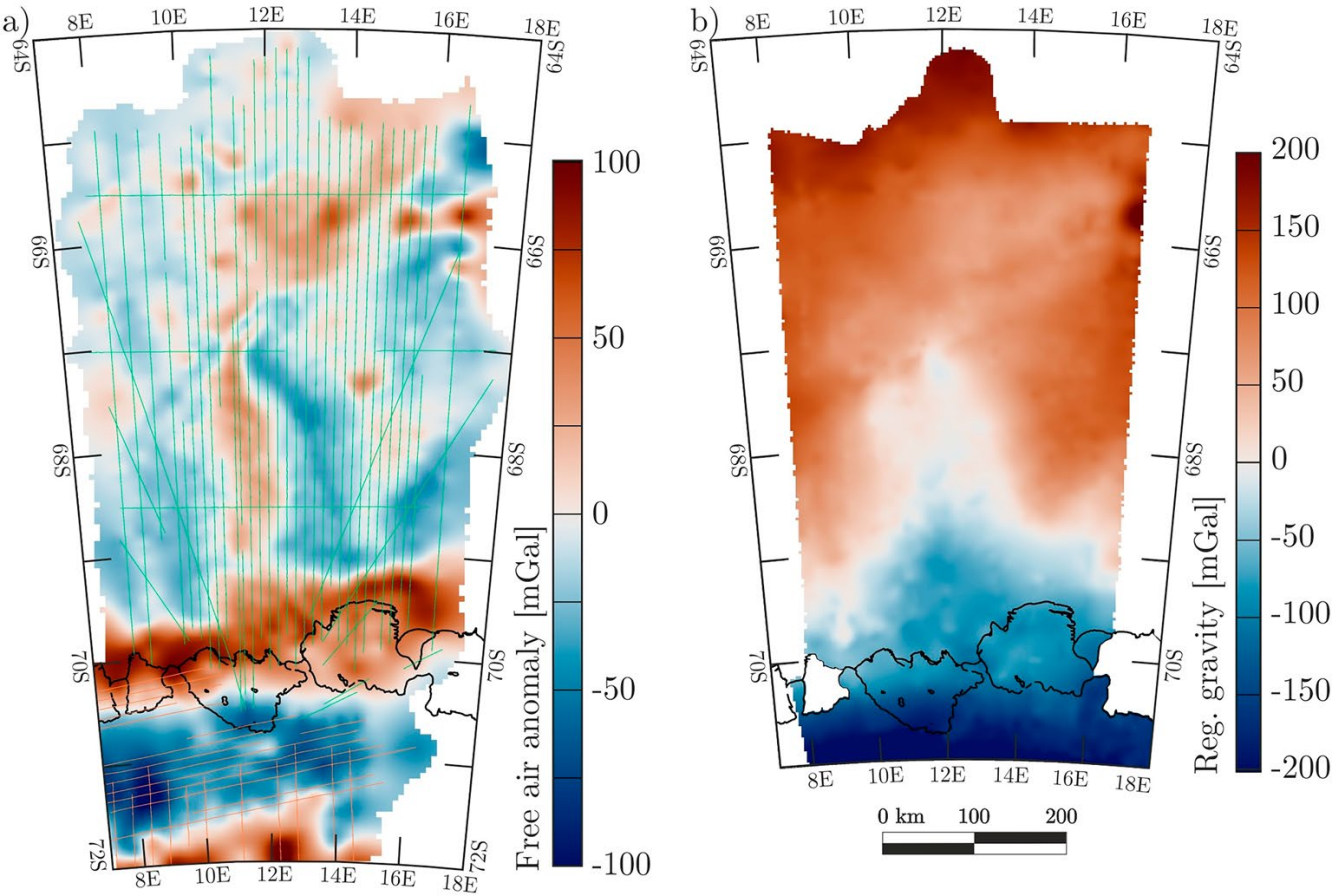
Free air anomaly data and regional gravity field. (**a**) airborne gravity data from VISA^32^ (orange lines), and WEGAS campaigns (green lines). (**b**) shows the regional gravity field. Here, gravity residuals between calculated and observed gravity data at points of known bathymetry/topography are interpolated across the entire model area including areas of unknown topography. This regional field is subtracted from the observed airborne gravity data in (**a**). Resulting gravity anomalies are then inverted for bathymetry/subglacial topography. Neighbouring Vigrid and Borchgrevink ice shelves are overlain in white. The scale is valid for both (**a**) and (**b**). Calving fronts and grounded areas are extracted from MEaSUREs data collection^18^. Figure is generated with *Seequent’s Geosoft Oasis montaj* and *Corel Draw*.

The large gaps can be closed by bathymetry inferred from airborne gravity data (Fig. 4). Our gravity data were acquired during the campaign West–East Gondwana Amalgamation and its Separation (WEGAS) in the austral summer of 2009/2010 with 21,000 line-km^21^. And in the early 2000’s as part of the multi-year VISA campaign (Validation, densification and Interpretation of Satellite data for the determination of magnetic field, gravity field, ice mass balance and structure of the Earth’s crust in Antarctica using airborne and terrestrial measurements)^32^. The data comprise indirect constraints on bathymetry with along-track resolution of ~ 7 km. More detailed descriptions of available topographic data sets, airborne gravity data across the survey region, and its compilation are supplied in the methods section.

## Results

**Figure 5:**
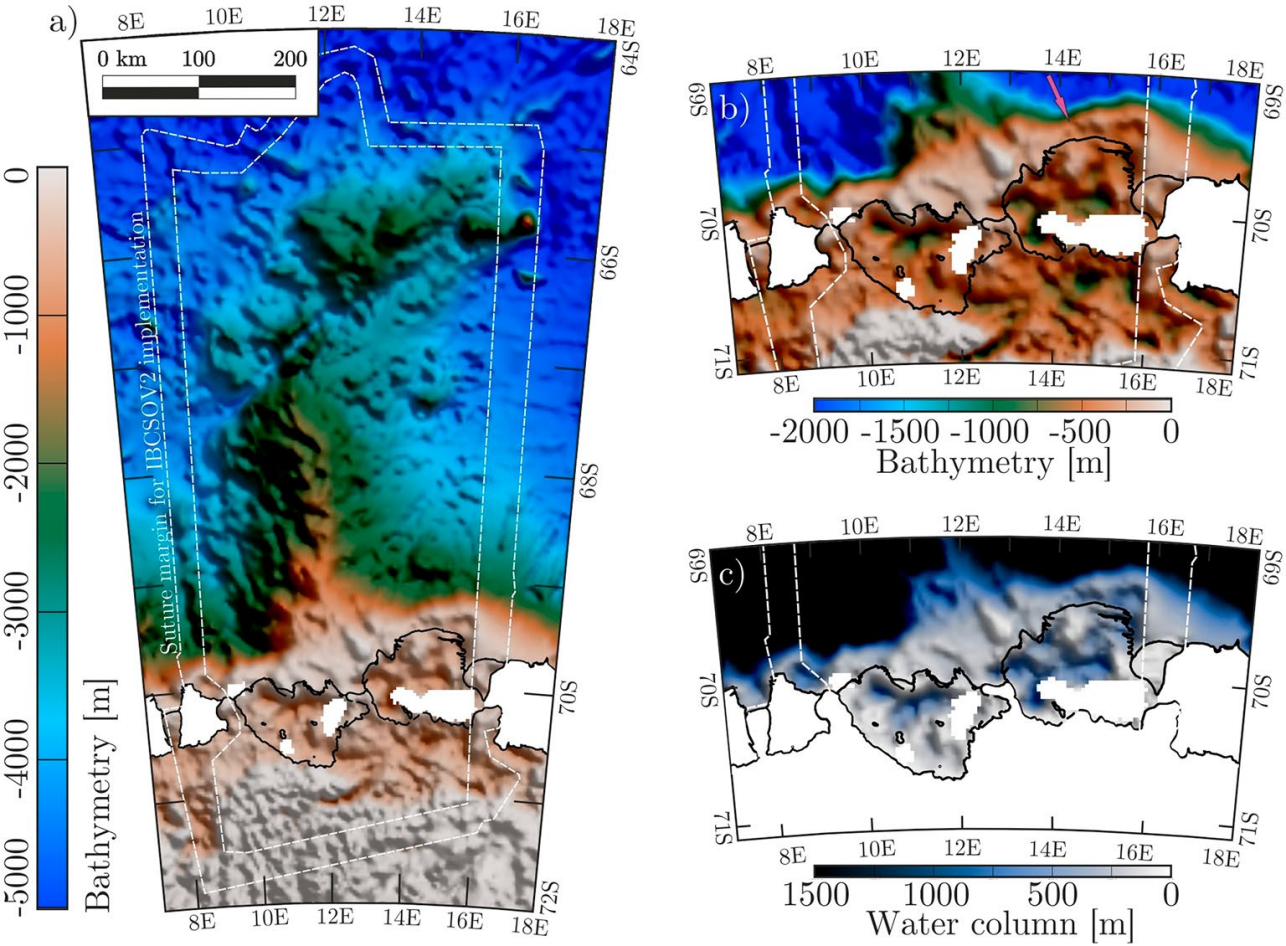
Bathymetric model of Astrid Ridge, Nivl Ice Shelf and Lazarev Ice Shelf. (**a**) shows bedrock topography in model area, gridded with 2500 m cell size, blanking distance of 5000 m and cell extension of 1. The model is smoothly merged with IBCSO V2^12^ using an overlap marked with white dashed lines. (**b**) shows a detailed view of the Nivl and Lazarev ice shelves with an adapted colour scale. (**c**) shows water column thickness (cavity height) between the bedrock and the ice base/sea surface.. Neighbouring Vigrid and Borchgrevink ice shelves are masked. Calving fronts and grounded areas are extracted from MEaSUREs data collection^18^. Figure is generated with *Seequent’s Geosoft Oasis montaj* and *Corel Draw*.

The bathymetric model in Fig. 5a shows depths in metres, relative to the WGS84 ellipsoid, for the region covering the Nivl and Lazarev ice shelves and the offshore Astrid Ridge. It was generated utilizing available topographic data sets (Fig. 2) and by inverting available gravity data (Fig. 4a). Figure 5b gives a closer view of the model over the Nivl and Lazarev ice shelves. Figure 5c shows the underlying water column thickness, calculated from the difference between the bedrock and the ice base/sea surface. The incomplete coverage of available airborne gravity data leaves small gaps in the model over the two ice shelves.

The bathymetric model was generated using the extension *GM-SYS 3D Research* of *Seequent’s Geosoft Oasis montaj*. Within this module, a calculation of the laterally varying regional gravity field (Fig. 4b) was utilized in inferring the bathymetry. The estimated error of our model lies within the range of about 138 to 160 m. A more detailed elaboration on the bathymetric modelling process, and our error estimation is given in the methods section.

### Comparison to IBCSO V2

IBCSO V2^12^ is the most comprehensive available compilation of shipborne bathymetric data offshore, and is seamlessly combined with the latest version of the BedMachine Antarctica topographic compilation for areas beneath grounded ice and ice shelves^15^. Neither compilation uses any of the seismic constraints of Fig. 2, and both instead depict BedMachine Antarctica’s depths, which merely allow for the radar-measured Nivl and Lazarev ice shelf bases to float free of the seabed. North of 66 to 67° S, where the influence of sea ice diminishes, IBCSO V2^12^ incorporated bathymetry derived from satellite altimetry^11^. This results in a clear northward increase in the spatial resolution of topographic features (across the dashed white line in Fig. 1a).

**Figure 6:**
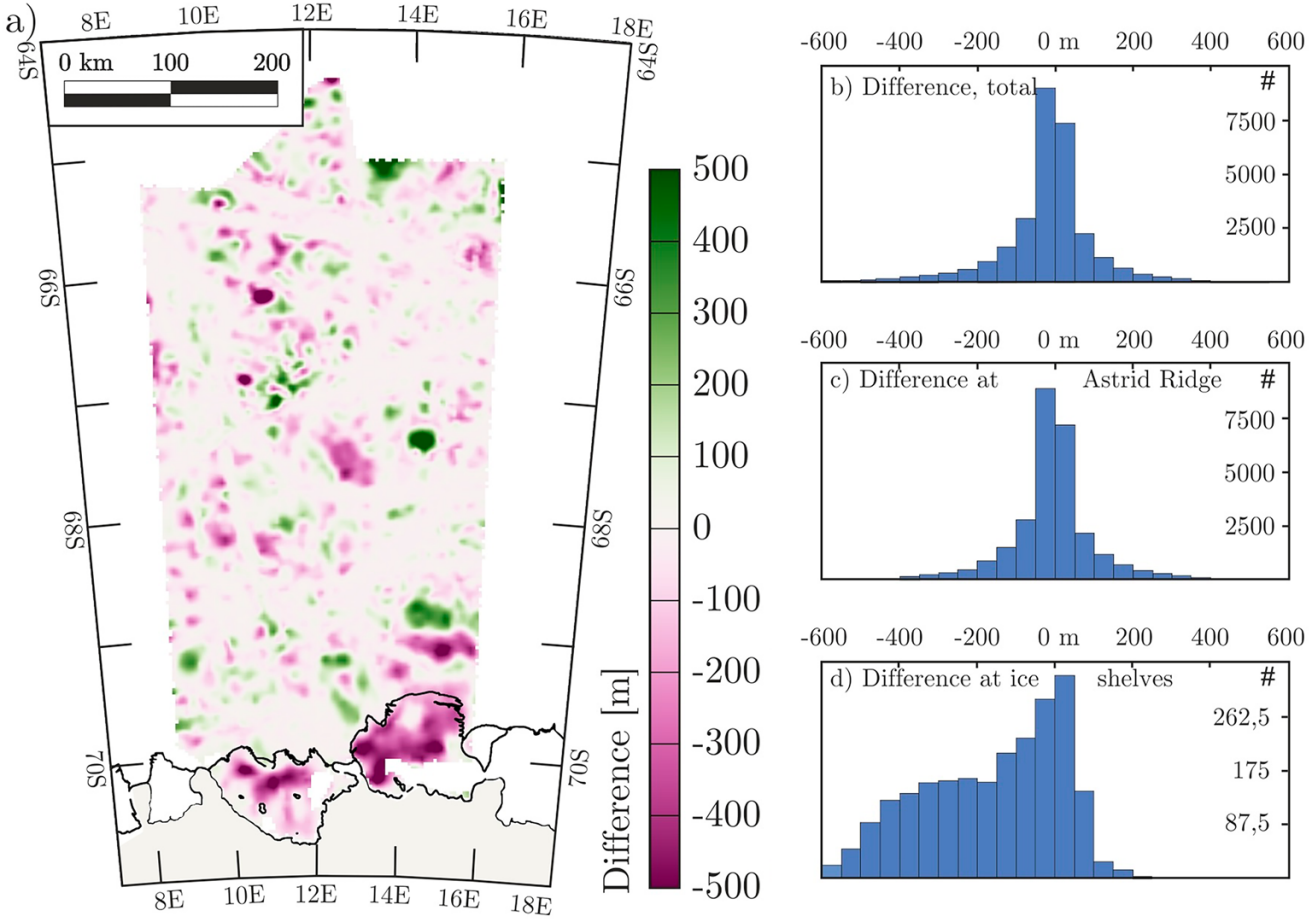
Comparison of aerogravity-based bathymetric model with IBCSO V2. Model in Fig. 5 is compared to most recent topography compilation IBCSO V2^12^ in (**a**). Histograms show the difference between the topography for (**b**) modelled area north of the grounding lines, (**c**) modelled area north of calving fronts, and (**d**) modelled area beneath Nivl and Lazarev ice shelves. Positive values indicate that the new bathymetric model values are shallower, negative values indicate deeper values compared to IBCSO V2. Calving fronts and grounded areas are extracted from MEaSUREs data collection^18^. Figure is generated with *Seequent’s Geosoft Oasis montaj* and *Corel Draw*.

**Figure 7:**
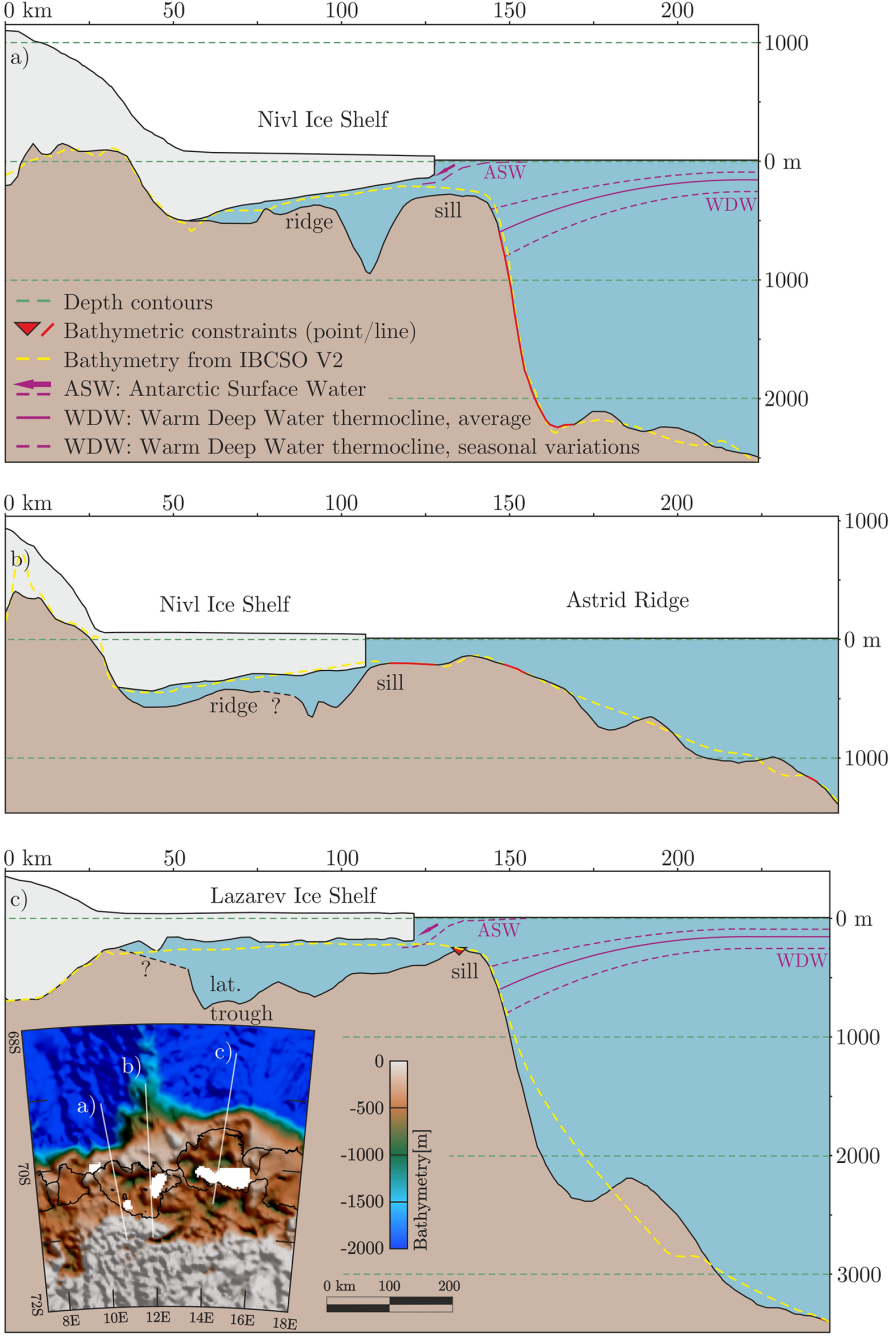
Two-dimensional cross sections of bathymetric model. (**a**) and (**b**) show two profiles along the Nivl Ice Shelf, while (**c**) shows one profile along the Lazarev Ice Shelf. Vertical exaggeration for all profiles is ~ 34. An overview map with the course of the three profiles is depicted in the bottom frame. Calving fronts and grounded areas here are extracted from MEaSUREs data collection^18^. Figure is generated with *Seequent’s Geosoft Oasis montaj* and *Corel Draw*.

Residuals of bathymetry and subglacial bathymetry depicted in our bathymetric model (Fig. 5a; grid spacing of 2500 m) and that of IBCSO V2 (Fig. 1; resolution of 500 m) are shown with histograms for individual areas (Fig. 6). Despite the differing spatial resolutions, our model shows considerably more detail, especially in the so-called ‘transition zone’ of IBCSO V2 where satellite-derived seabed depths^11^ were not integrated. The modelled seabed overall has a mean difference of − 19 m to IBCSO V2 with a standard deviation of 128 m (Fig. 6b). The largest differences are those beneath the two ice shelves, where our model shows the seafloor to be considerably deeper than in IBCSO V2. This is especially evident when comparing histograms for the modelled area offshore (Fig. 6c), and the modelled area beneath ice shelves (Fig. 6d). In the latter case, the mean error lies at − 168 m with a standard deviation around this mean of 180 m. As a consequence, the volume of the Nivl Ice Shelf cavity, estimated at just 278 km^3^ in IBCSO V2, increases to 1069 km^3^ in our better-constrained model. The volume of the Lazarev Ice Shelf increases from 390 km^3^ in IBCSO V2 to 2,007 km^3^ in our model. This underestimation of cavities is underlined by three two-dimensional profiles in Fig. 7.

Differences between the new model and IBCSO V2 bathymetries further offshore are more balanced. The mean difference is −7 m and the standard deviation of differences is 115 m. Systematic differences can be observed over the continental shelf at the southern end of Astrid Ridge, and over both of the ridge’s flanks, all of which are depicted in much more detail in the new model (Figs. 1, 5 and 6). A comparison of our bathymetric model to the correlation-derived SRTM15 + bathymetry^11^ in the offshore area returned a mean error of -10 m and a standard deviation of 175 m. The significantly broader distribution than in the comparison to IBCSO V2 is explained by the more limited ship-based dataset used for SRTM15+^11^.

### Nivl and Lazarev ice shelves

Seafloor beneath the Nivl Ice Shelf is characterized by two latitudinal ridges. The more seaward ridge runs along 70° S beneath the calving front (Figs. 5, 7, bathymetric sill), with culminations forming four separate pinning points. Between them, the seabed is relatively shallow with a mean value of about 200 m and water column thickness not exceeding 170 m. There is no meaningful gateway through the sill connecting the cavity to the north with the open ocean.

South of the seaward ridge, the seabed deepens to as much as 950 m (Figs. 5 and 7). South of this, a second ridge connecting three pinning points runs roughly along 70.4° S (Fig. 7; bathymetric ridge). The southern seismic reflection line shows a local high between the two ridges (Fig. 3b, at km 7 to 8), where the water column shallows to about 100 m (Fig. 3b). In general, the southern seismic line shows seabed reflections with no penetration, except one section of well stratified reflections at km 4 to 6 beneath the depression between sill and ridge (Fig. 3b). The modelled seabed is quite shallow between the southern ridge and the grounding line, with water column heights not exceeding 250 m (Fig. 5). However, here the model is based on one line of gravity data and three seismic reflection points only (Fig. 2).

Bathymetry beneath the Lazarev Ice Shelf shows stronger variations. The seabed beneath the eastern calving front ranges in depth between 250 and 550 m, shallowing westwards. The deepest points are flanked by shallower bathymetry further south beneath the ice shelf. A possible gateway into the open ocean is modelled with a minimum depth of 370 m (pink arrow in Fig. 5b). Mean depths of around 250 m characterise the seafloor further west beneath the calving front and in the area south of it to 69.8° S. At its westernmost end, however, this pattern is broken by the previously mapped 700-m deep depression known as Fenno Deep^37^. Shipborne bathymetric data show that the deep is not part of a continuous seafloor trough beneath the calving fronts of the Lazarev and Nivl Ice shelves (Fig. 2b). Our model reproduces this observation (Fig. 5), and shows further that the deep is the westernmost point along a deep latitudinal trough that continues beneath the entire ice shelf apart from near its eastern edge (Figs. 5 and 7c). Depths in the trough reach up to 900 m with a mean value of 700 m. The deepest point beneath the Lazarev Ice Shelf lies at 1100 m, below its southwestern corner. Small areas in the south and southeast of the Lazarev ice shelf lack either seismic or gravity data (Figs. 2 and 4) and are thus not modelled (gaps in Figs. 5, 7c).

### Astrid Ridge and offshore

The shallow depths beneath and close to the calving fronts of both the Nivl and the Lazarev Ice Shelf occur on a broad continental shelf (Figs. 5 and 7b); an exception to the usually narrower shelf of Dronning Maud Land caused by the presence of the Astrid Ridge. This elongated feature lies orthogonal to the continental shelf and projects northwards of it for 500 km, gently deepening, until about 65° S. The existing shipborne bathymetric measurements across the ridge, which are relatively sparse north of 68.5° S, are now complemented by inverted-for bathymetry based on gravity data (Fig. 5). The new model clearly shows a step-like transition between the continental shelf edge and the offshore part of Astrid Ridge that is not evident in the IBCSO V2 grid (Figs. 1b, 2).

Astrid Ridge narrows northwards away from the continental shelf edge towards its termination (Figs. 5 and 7b). The wider southern part (south of 67° S) is flanked on its eastern and western margins by canyon-like structures on steep slopes. The new model presents a distinct circular seamount close to 15° E/67° S. Rising to a depth of 2700 m above adjacent seafloor of 3600 m, the seamount is depicted around 900 m shallower than by interpolation in IBCSO V2, but 500 m deeper than by SRTM15+^11^.

## Discussion

Utilising existing topographic data and available airborne gravity data, we have generated an update to existing bathymetric knowledge in an area of around 210,000 km^2^ beneath the Nivl and Lazarev ice shelves of central Dronning Maud Land, and over the Astrid Ridge, and in the Riiser-Larsen Sea further offshore. This process was aided by the recovery of an unpublished seismic reflection line across the Nivl Ice Shelf^35,36^ (Figs. 2 and 3) and seismic reflection point data across both ice shelves^33^ (Fig. 2), none of which are included in recent topographic compilations.

The largest differences of our model to the current topographic knowledge (e.g., IBCSO V2^12^) are found beneath the Nivl and Lazarev ice shelves (Figs. 6 and 7). These differences are significant at the level of accuracy of the gravity inversion method used, and imply the cavity volume to be 480% greater than previous data sets have shown. This figure is subject to change as small areas remain to be adequately depicted owing to insufficient gravity data coverage (Fig. 5a).

### Bathymetric patterns and glacial history

The subglacial bathymetry at the ice shelves of Dronning Maud Land and Coats Land reveal some consistent themes that recur in the new data set. Foremost among these is the bathymetric sill observed to run nearly continuously along or near the continental shelf break (Fig. 7). As well as beneath the Nivl and Lazarev ice shelves, further parts of the sill can be observed at the Brunt Ice Shelf and Stancomb-Wills Glacier Tongue^38^, the Ekström, Jelbart, Fimbul and Vigrid ice shelves^8^, and at the Borchgrevink and Roi Baudouin ice shelves^24^. All of the ice shelves are locally grounded at pinning points, ice rises, and islands dotted along this sill^18^. We interpret the sill as being underlain by moraines. As such, it serves as direct observational evidence in support of proposals^39,40^ that the East Antarctic Ice Sheet advanced up to the continental shelf edges of Coats Land and Dronning Maud Land during the Last Glacial Maximum (23–19 kyr BP). At distinct locations beneath many of the ice shelves, the shelf-edge sills are interrupted by deeper gateways leading into the cavities, potentially allowing the ingress of Warm Deep Water^8,24^. Beneath the Nivl and Lazarev ice shelves, however, no significant gateways were identified (Fig. 5).

Behind their sills, the floors of ice shelf cavities in Dronning Maud and Coats Land deepen into troughs that lead up towards the grounding line. For most of these ice shelves, the deepest troughs mirror the traces of fastest flow in the overlying shelf, and so may well be overdeepenings and thus, erosional in origin^8,24,38^. Consistent with this pattern, the seafloor beneath the Nivl and Lazarev ice shelves also deepens significantly south of the bathymetric sill (Figs. 5 and 7). However, the incomplete gravity data coverage over the shelves means we cannot definitely state that these troughs continue up towards the grounding line and/or might represent continuations of troughs onshore (e.g., Potsdam Glacier trough in Fig. 5). If the troughs are not continuous, it is possible that they could result in a compartmentalization of circulation regimes.

The presence of glacial troughs and terminal moraines along the entirety of this part of the Antarctic margin suggests a common origin. At neighbouring ice shelves, the glacial troughs continue their path into gateways along the sill^8,24^, giving rise to the idea that the gateways are also a consequence of former trough continuations. If this is the case, gateway depths at sills could be related to the vigour of former ice sheet/shelf flow. Consequently, in areas of less vigorous ice flux, no distinct gateways into the cavities are formed, as is the case for the Nivl and Lazarev ice shelves (Fig. 5).

Generally, the glacial troughs beneath the ice shelves of Dronning Maud and Coats Land appear to be segmented by minor bathymetric ridges that run parallel to the continental shelf and perpendicular to present ice flow. These patterns are observed beneath the Brunt Ice Shelf and Stancomb-Wills Glacier Tongue in Coats Land^38^, the Ekström, Jelbart and Fimbul ice shelves^8^, and the Borchgrevink and Roi Baudouin ice shelves^24^. Shelf-parallel ridges are also present beneath both the Nivl and Lazarev ice shelves, culminating in a chain of pinning points beneath the Nivl Ice Shelf and rapid shallowing northwards at 69.8° S beneath the Lazarev Ice Shelf. In all of these settings, the ridges appear to be related to deposition at grounding zones during phases of readvance within the last glacial cycle, as postulated for the Weddell Sea sector^41^.

### Ice–ocean interactions

The Weddell Gyre transports Warm Deep Water, Antarctic Surface Water, and Eastern Shelf Water along the coast of Dronning Maud Land. The gyre extends to about 30° E^42^, where an elongated topographic high, Gunnerus Ridge, steers the inflow of Circumpolar Deep Water of the Antarctic Circumpolar Current into the gyre system^23^.

Astrid Ridge, at 10° E to 15° E, separates the easterly Riiser-Larsen Sea from the westerly Lazarev Sea at varying seabed depths from 200 m at its transition to the continental shelf to more than 2000 m further offshore. The average thermocline depth of Warm Deep Water lies at about ~ 600 m both east and west of the ridge off the Roi Baudouin Ice Shelf^43,44^, and the Fimbul and Riiser-Larsen ice shelves^7,45^, suggesting it has little influence on the large-scale structure of the gyre. The local structure of the thermocline and patterns of coastal water flow and mass exchange to the Nivl and Lazarev ice shelves over the ridge are underexplored. However, seasonal variations of thermocline depth have been observed in coastal Dronning Maud Land with a shallowing thermocline in spring/summer and deepening in the winter^45^.

The new bathymetric model shows that the Nivl Ice Shelf is quite isolated by its outer bathymetric sill, which lacks any notable gateway into the cavity. This, together with the lack of lateral deep connections to the neighbouring cavities, suggests that the Nivl Ice Shelf does not experience inflow of Warm Deep Water, even assuming a summer thermocline depth as shallow as 400 m (Fig. 7). Although the Lazarev cavity’s sill is crossed by a deeper gateway, at ~ 370 m deep, this is still considerably shallower than regional or seasonal thermocline depth^7,43,45^. Given current knowledge of the thermocline, therefore, significant Warm Deep Water intrusion is unlikely here as well.

Since both ice shelf cavities are not, or at most only sporadically^7^, accessible to Warm Deep Water, basal melting processes are likely dominated by freshened and solar-heated Antarctic Surface Water (Fig. 7). Intrusions of this water only occur seasonally as a response to the combination of easterly winds, the presence of a fairly narrow continental shelf, and sea-ice free summer months^26^. Across the Nivl Ice Shelf, the likelihood of this process is supported by observations of strong seasonal variations in basal melting using phase-sensitive radar data across the Nivl Ice Shelf^25^. The Lazarev Ice Shelf lacks comparable data, but its only slightly deeper bathymetric setting suggests that basal melting there may also be dominated by seasonal intrusions of solar-heated water.

The contrasting bathymetric settings of the Nivl and Lazarev ice shelves suggest they might react in contrasting ways to possible near and mid-term warming of the Southern Ocean^46,47^, and/or changes in thermocline depth. With its multitude of pinning points along two latitudinal ridges, the Nivl Ice Shelf is buttressed well and could long withstand rising Warm Deep Water depth by blocking its ingress. The Lazarev Ice Shelf, however, is buttressed by just three pinning points, all situated in its western sector. This weaker and spatially inhomogeneous buttressing pattern is mirrored today in much larger ice flow velocities for the Lazarev Ice Shelf, especially in its east (Fig. 1b). As well as this, as the Lazarev cavity’s outer sill lies somewhat deeper than Nivl’s, future ingress of shallower Warm Deep Water, seasonally or year-round, can be expected to be greater, and to start sooner, than into the Nivl cavity.

Our bathymetric model provides a crucial boundary condition for regional oceanographic models to further investigate the present and future stabilities of the Nivl and Lazarev ice shelves, as well as the role of the Astrid Ridge in the exchange of water masses between ice shelf cavities and the open ocean.

### Bathymetric knowledge gaps along Antarctica’s margin

Within this paper, we underline the importance for knowledge of seabed topography along Antarctica’s margins to assess ice-ocean interactions; not only beneath the coastal ice shelves—a necessity fairly well understood and worked on in recent time –, but also in areas covered by sea ice year-round. Here, the problems related to sparse availability of bathymetric measurements and lowered accuracy of satellite altimetry-based predictions are equally serious.

Our new model shows that inversion of airborne gravity data offers great potential to rapidly map bathymetry in areas of multi-year sea ice, like that over the southern part of Astrid Ridge. A comparison to IBCSO V2 shows that the inversion technique delivers abundant details that interpolations between widely spaced soundings cannot. Conservative forwards consideration of the accuracy of the technique suggests these detailed depths may be accurate to around 160 m, comparable to the 150–180 m figures suggested for gravity-correlation techniques over the ice-free areas of the open ocean and continental shelves^11^. To evaluate this comparison further, we compared an early iteration of our bathymetric model to single-beam measurements that had yet to be incorporated as constraints on the inversion, observing a root mean square error of 138 m.

Prior to our study, the gravity inversion method was also applied to a smaller survey area around and offshore of the Cook Ice Shelf and Ninnis Glacier Tongue^48^. The two studies show that the method of inverting near-surface gravity data, which is frequently and increasingly used to model the seabed beneath ice shelves, can also be applied to underexplored areas of the open ocean as well, with results of improved accuracy over those of the correlation-based method^11^. This would be of particular significance for any of the ‘transition zone’ region’s around Antarctica’s margins where IBCSO V2^12^ did not incorporate bathymetry from SRTM15+^11^ and where systematic airborne gravity data are available. The largest of these ‘transition zones’ in IBCSO V2^12^ is in the Weddell Sea Embayment, where around 0.5 million square kilometres of the seabed remain bathymetrically unobserved. Existing and targeted airborne gravity surveys might offer the most realistic prospect of charting these areas at resolutions suitable for climate and oceanographic models.

## Methods

### Topographic data

Inputs into our bathymetric model consist of topographic information about the ice surface, ice base and bedrock topography. Surface heights are extracted from REMA^16^ in ice-covered regions and from Bedmap^13^ for the ellipsoidal height of the open ocean, while the positions of grounding lines, calving fronts and pinning points are extracted from the MEaSUREs data collection^18^.

For the most part, bedrock topography over areas of grounded ice surrounding the Nivl and Lazarev ice shelves is incorporated into the bathymetry model on the basis of ice penetrating radar data^32^ (Fig. 2). These radar data also cover a portion of the pinning points across the two ice shelves. A pinning point in the NW of the Nivl Ice Shelf is additionally covered by ground-based radar data^25^. Remaining gaps regarding the ice base, including remaining pinning points, are closed by integrating the ice base from seismic reflection data described below and from BedMachine Antarctica^15^.

At the calving fronts and offshore of them across the Astrid Ridge, the model incorporates multibeam data from several research cruises^27–31^, as well as single-beam data all incorporated into IBCSO V2^12^.

Bathymetric knowledge beneath the Nivl and Lazarev ice shelves is presently limited to the results of seismic depth soundings. By Antarctic standards, these soundings are plentiful in relation to the ice shelves’ sizes, potentially making their cavities two of the best explored cavities anywhere in Antarctica. To date, however, these soundings have not been compiled for a complete picture of either cavity.

Seismic point reflection data with detection of the seabed were successfully acquired at 130 locations across the Lazarev Ice Shelf and in its vicinity (yellow circles in Fig. 2a) during Soviet expeditions^34^. These point data are already included in the upcoming Bedmap3 compilation^14^. Additional seismic point soundings were acquired during the GeoMaud expedition in 1995/96^49^. Seismic data were acquired at six sites across the Nivl Ice Shelf and two sites at the Lazarev Ice Shelf using a ‘Sissy’ seismic gun and a Geometrics 2401 instrument with 24 channels^33^ (green circles in Fig. 2a). These soundings have not yet been included in any topographic compilation.

The GeoMaud expedition also saw the University of Münster, Germany, acquire seismic reflection profiles over the NE of the Nivl Ice Shelf. These data were never published outside of diploma theses^35,36^. The profiles were acquired with explosives, delivering a tenfold southern seismic line of 9.5 km and a single-fold northern profile of 9 km (red and orange lines Figs. 2a, 3). The southern line was acquired with 0.9 kg and 2.1 kg of explosives for the near- and far-shot with an array of 60 geophone groups with 18 m distance, while the northern line was acquired with a simpler single receiver setup, but a larger explosive charge of 15 kg (M. Degutsch, pers. comm.). Unfortunately, the hard data copies of both seismic profiles seem to be lost. Here, printouts of seismograms of the southern line^36^, and an interpreted section of the northern line^35^ are recovered and, in the latter case, modified visually (Fig. 3). The northern and southern profiles are contiguous and follow the same N–S heading. Measured and inferred parameters of both lines are given in the Supplementary Information (Table S1). Here, ice base and seabed depths are extracted from every 500 m in the southern profile, and in marked instances of the northern profile. The general trajectory of the profiles plus two shot locations were known at arc-minute accuracy^35,36^. These are used to extrapolate the positions of the two profiles in Table S1. Due to the lack of precise positioning information and the subsequent extrapolation, the horizontal accuracy is conservatively estimated to lie at around 2 km. A depth uncertainty interval of 25 m is given for the single-fold (northern) profile^35^, while no error interval for the ten-fold line is given. Although it is likely smaller than for the single-fold line, we conservatively estimated the same error interval, of 25 m.

### Gravity data

The VISA and WEGAS gravity anomaly data were all collected using the same ZLS Ultrasys modified LaCoste Romberg Air/Sea gravimeter (S/N 56), installed and operated on a gimbal stabilized platform. While the VISA data were acquired at a high flight level of about 3 to 4 km, the WEGAS data were acquired offshore, allowing a constant flight level of around 200 m, and so are better suited for an inversion towards topography. A half power point of 200 s with a ground speed of 140 knots results in a horizontal resolution of about 7 km for both data sets^24,32^. Based on cross-point analyses, root mean square errors of 4.3 mGal are. reported for the VISA data^32^, while the equivalent figure for the WEGAS data set is 4.1 mGal.

Gravity data from the two campaigns do not intersect (Fig. 4). To combine them in one consistent grid, we have implemented a previously developed approach^50^ in which the potential offsets of individual surveys to the regional satellite gravity field are calculated and minimized. Here, the available airborne gravity data from survey campaigns WEGAS and VISA are referenced to the satellite gravity field GOCO06s^51^. In detail, all three data sets are filtered with a low-pass filter of 200 km to enable the comparisons. The filtered WEGAS data returned a mean residual of 0 mGal to GOCO06s, while the filtered VISA residual was around 5 mGal. To establish a zero-mean offset to GOCO6s, therefore the unfiltered WEGAS gravity data were adopted without change, and the initial VISA gravity data were shifted by 5 mGal. Resulting line data from WEGAS and VISA were then gridded with a cell size of 5 km, as shown in Fig. 4.

### Bathymetry modelling

Bathymetry modelling is conducted using the extension *GM-SYS 3D Research* of *Seequent’s Geosoft Oasis montaj*, similar to the approaches of similar studies^8,24^. The module implements previously introduced formulas for gravitational acceleration^52,53^.

The model has a maximum depth of 10 km and a horizontal grid spacing of 2.5 km. This is less than the inferred horizontal resolution of 7 km for the gravity data, as described above, but it is locally appropriate to the level of detail in known bathymetry inputs, which would otherwise not be captured with a less-detailed resolution. The topographic model consists of three different interfaces, one each at the ice surface, ice base, and bedrock topography. Layers between these interfaces are assigned densities for ice (917 kg/m^3^), sea water (1028 kg/m^3^) and bedrock (2670 kg/m^3^). The observation plane is adapted to the differing acquisition heights of the two surveys.

First, the three topographic interfaces consisting of ice surface, ice base, and bedrock topography, are integrated into the model domain with the distinct densities mentioned above. A gravity inversion is performed to calculate the differences in observed gravity anomalies from airborne surveys and calculated gravity anomalies from the model. The observed and calculated gravity anomalies are compared and then subtracted at points of known topography. These residuals are subsequently used as the basis for interpolation across the whole survey area and represent the regional gravity field (Fig. 4b). This regional gravity field is subtracted from the initial observed gravity data input and an inversion towards bedrock topography is performed with this result.

For the inversion, the bedrock surface for grid points that include locations with soundings is allowed to move within an envelope of ± 25 m, a reasonable assumption in the face of expected errors in airborne ice penetrating radar data^54^ and seismic data across the shelf.

### Error estimation

The accuracy of our bathymetric model is determined by instrumental and methodological limitations. First and foremost, the accuracy of all seismic data sets across the ice shelves follows the proposed vertical accuracy of ± 25 m^35^. To keep consistency for known bedrock topography, the accuracy of ice penetrating radar data is conservatively estimated to lie at ± 25 m as well^54^.

As described in Sect. 2.2 in the main text, a mean crossover error of 4.3 mGal for VISA campaigns was reported^32^, while the WEGAS data set has a root mean square error of 4.1 mGal. The error evaluation for the bathymetric model is based on the larger error of 4.3 mGal. After successful bedrock inversion, the resulting gravity residuals in the model area—excluding the grounded ice sheets—have a root mean square error of 0.4 mGal (Fig. S1, see Supplementary Information). Maximum values of 5 mGal are registered close to the Kuvklaken pinning point at the Nivl Ice Shelf with similarly high residuals west of the Astrid Ridge. In the first case, a shallower bathymetry is pursued during the modelling process but prevented by the prevalent ice base and thus, the gravity residuals surge. In areas of open ocean, highly undulating topography results in the same surge of gravity residuals^24^.

Generally, the density contrast between water and bedrock (1642 kg/m^3^) has greater influence on the final model than the density contrast between water and ice (111 kg/m^3^). A bouguer slab calculation using the combined crossover errors of the initial gravity data sets (4.3 mGal) and the maximum gravity residual after model completion (5 mGal) and density contrast of 1642 kg/m^3^ implies errors of up to ± 135 m in the modelled bathymetry. Together with the errors associated with ice penetrating radar and seismic data (± 25 m), the overall estimated error envelope is ± 160 m for a model resolution of 2.5 km.

A slightly varying error estimation can be based on a comparison of first modelling results to available depth references. In a first iteration of the bathymetric model generation, the network of single-beam data available in IBCSO V2 was not included as topographic constraints during the modelling process. The regional gravity field at these points was interpolated from neighbouring constraints and the model seabed was thus allowed to move freely. When comparing modelling results to the single-beam data, a root mean square error of 138 m was observed. The error of our bathymetric model is thus estimated to lie within the range of 138 to 160 m.

### Supplementary Information

Supplementary Information.

## Data Availability

Both airborne gravity data and the bathymetric model are uploaded to the public data repository PANGAEA here: Gravity data (10.1594/PANGAEA.961497); Bathymetric model: (10.1594/PANGAEA.961492); Bathymetric model embedded into IBCSO V2: (10.1594/PANGAEA.963737).
