# Supplementary material for: Coastal bathymetry in central Dronning Maud Land controls ice shelf stability
Source: Sci Rep. 2024 Jan 16;14:1367. doi: 10.1038/s41598-024-51882-2 (PMC10791693; doi:10.1038/s41598-024-51882-2)
Supplement: Supplementary file 1 — Supplementary Information. [file 41598_2024_51882_MOESM1_ESM.docx]

**Supplementary Information:**

**Coastal bathymetry in central Dronning Maud Land controls ice shelf stability**

Eisermann, H.^1^, Eagles, G.^1^, & Jokat, W.^1,2^

^1^Alfred Wegener Institute, Helmholtz Centre for Polar and Marine Research, Bremerhaven, Germany

In the following order, the Supplementary Information consists of

1. Table S1: Seismic Profiles across the Nivl Ice Shelf
2. Figure S1: Gravity residuals after successful inversion

# Seismic Profiles

**Table S1: Seismic profile along the NE part of Nivl Ice Shelf with its location and registered horizon depths.** Positions of shot points are interpolated and extrapolated from information on two locations and the general trajectory of the profiles. Horizon depths are extracted from marked instances in the northern profile and inferred from the seismogram of the southern profile.

| **Northern Profile** | | | | | |
| --- | --- | --- | --- | --- | --- |
| **Longitude [WGS84]** | **Latitude [WGS84]** | **Northern Profile [km]** | **Ice Base [m]** | **Seabed [m]** | **Comment** |
| 11.739659 | -70.080502 | 9.70 | -232.00 | -291.0 | extrapolated |
| 11.739941 | -70.083184 | 9.40 | -232.00 | -315.0 |  |
| 11.740318 | -70.086760 | 9.00 | -232.00 | -347.0 |  |
| 11.740412 | -70.087654 | 8.90 | -232.00 | -361.0 |  |
| 11.740601 | -70.089441 | 8.70 | -244.00 | -380.0 |  |
| 11.740789 | -70.091229 | 8.50 | -244.00 | -400.0 |  |
| 11.741260 | -70.095699 | 8.00 | -244.00 | -412.0 |  |
| 11.741637 | -70.099274 | 7.60 | -244.00 | * | *-394 m or -415 m |
| 11.742298 | -70.105532 | 6.90 | -244.00 | -423.0 |  |
| 11.742675 | -70.109108 | 6.50 | -244.00 | -440.0 |  |
| 11.742864 | -70.110896 | 6.30 | -242.00 | -423.0 |  |
| 11.743148 | -70.113578 | 6.00 | -241.00 | -447.0 |  |
| 11.743336 | -70.115366 | 5.80 | -241.00 | -471.0 |  |
| 11.743620 | -70.118048 | 5.50 | -250.00 | -500.0 |  |
| 11.743809 | -70.119836 | 5.30 | -250.00 | -512.0 |  |
| 11.744754 | -70.128775 | 4.30 | -250.00 | -618.0 |  |
| 11.745511 | -70.135928 | 3.50 | -250.00 | -634.0 |  |
| 11.745795 | -70.138610 | 3.20 | -251.00 | * | *-670 m or -744 m |
| 11.745890 | -70.139504 | 3.10 | -256.00 | -670.0 |  |
| 11.746269 | -70.143080 | 2.70 | -250.00 | -666.0 |  |
| 11.746458 | -70.144868 | 2.50 | -250.00 | -670.0 |  |
| 11.746838 | -70.148444 | 2.10 | -250.00 | -650.0 |  |
| 11.747786 | -70.157385 | 1.10 | -250.00 | -658.0 |  |
| 11.747976 | -70.159173 | 0.90 | -250.00 | -593.0 |  |
| 11.748830 | -70.167220 | 0.00 | -250.00 | -589.0 | known position |
| **Southern Profile** | |  |  |  |  |
| **Longitude [WGS84]** | **Latitude [WGS84]** | **Southern Profile [km]** | **Ice Base [m]** | **Seabed [m]** | **Comment** |
| 11.748830 | -70.167220 | 9.50 | -245.00 | -592.0 | known position |
| 11.749216 | -70.171684 | 9.00 | -247.00 | -559.0 |  |
| 11.749602 | -70.176149 | 8.50 | -244.00 | -482.0 |  |
| 11.749988 | -70.180613 | 8.00 | -235.00 | -425.0 |  |
| 11.750375 | -70.185078 | 7.50 | -254.00 | -418.0 |  |
| 11.750762 | -70.189543 | 7.00 | -256.00 | -441.0 |  |
| 11.751148 | -70.194007 | 6.50 | -259.00 | -557.0 |  |
| 11.751535 | -70.198472 | 6.00 | -265.00 | -667.0 |  |
| 11.751923 | -70.202937 | 5.50 | -271.00 | -703.0 |  |
| 11.752000 | -70.203830 | 5.40 | * | * | known position |
| 11.752323 | -70.207386 | 5.00 | -273.00 | -750.0 |  |
| 11.752726 | -70.211830 | 4.50 | -278.00 | -786.0 |  |
| 11.753129 | -70.216275 | 4.00 | -280.00 | -747.0 |  |
| 11.753533 | -70.220720 | 3.50 | -282.00 | -615.0 |  |
| 11.753937 | -70.225165 | 3.00 | -288.00 | -505.0 |  |
| 11.754341 | -70.229609 | 2.50 | -288.00 | -553.0 |  |
| 11.754745 | -70.234054 | 2.00 | -294.00 | -538.0 |  |
| 11.755149 | -70.238499 | 1.50 | -295.00 | -507.0 |  |
| 11.755554 | -70.242944 | 1.00 | -299.00 | -491.0 |  |
| 11.755959 | -70.247389 | 0.50 | * | * |  |
| 11.756364 | -70.251834 | 0.00 | * | * |  |
| 11.757580 | -70.265170 | * | * | * | extrapolated |


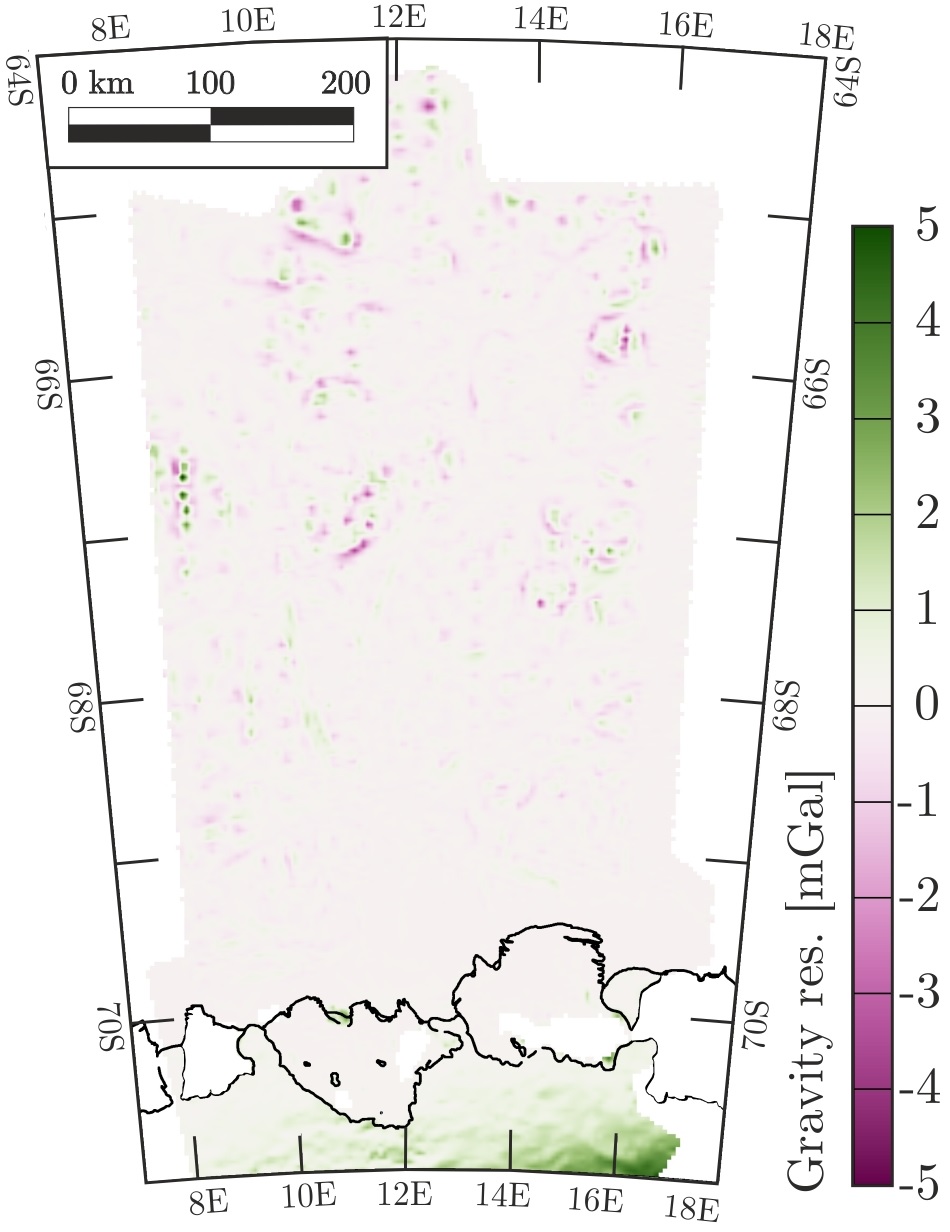


**Figure S1: Gravity residuals after modelling bathymetry in the region of the Nivl Ice Shelf, Lazarev Ice Shelf, and Astrid Ridge.** The calculation is limited to the extent of available gravity data. Residuals have a root mean square error of 0.4 mGal with individual peaks of up to 5 mGal. Neighbouring Vigrid and Borchgrevink ice shelves are overlain in white. Calving fronts and grounded areas are extracted from MEaSUREs data collection^1^. Figure is generated with *Seequent’s Geosoft Oasis montaj* and *Corel Draw*.

1. Mouginot, J., Rignot, E. & Scheuchl, B. MEaSUREs Antarctic Grounding Line from Differential Satellite Radar Interferometry, Version 2 [Data Set]. (2016) doi:10.5067/IKBWW4RYHF1Q.
